# Range-wide population genomics of common seadragons shows secondary contact over a former barrier and insights on illegal capture

**DOI:** 10.1186/s12915-023-01628-9

**Published:** 2023-05-29

**Authors:** Josefin Stiller, Nerida G. Wilson, Greg W. Rouse

**Affiliations:** 1 Scripps Institution of Oceanography, University of California San Diego, La Jolla, 92093 USA; 2 Centre for Biodiversity Genomics, University of Copenhagen, 2100 Copenhagen, Denmark; 3 Research & Collections, Western Australian Museum, Perth, Western Australia 6106 Australia; 4 School of Biological Sciences, University of Western Australia, Perth, Western Australia 6009 Australia

**Keywords:** Geogenetics, Vicariance, Secondary contact, Wildlife forensics, Syngnathidae, Common seadragon

## Abstract

**Background:**

Common seadragons (*Phyllopteryx taeniolatus*, Syngnathidae) are an emblem of the diverse endemic fauna of Australia’s southern rocky reefs, the newly recognized “Great Southern Reef.” A lack of assessments spanning this global biodiversity hotspot in its entirety is currently hampering an understanding of the factors that have contributed to its diversity. The common seadragon has a wide range across Australia's entire temperate south and includes a geogenetic break over a former land bridge, which has called its status as a single species into question. As a popular aquarium display that sells for high prices, common seadragons are also vulnerable to illegal capture.

**Results:**

Here, we provide range-wide nuclear sequences (986 variable Ultraconserved Elements) for 198 individuals and mitochondrial genomes for 140 individuals to assess species status, identify genetic units and their diversity, and trace the source of two poached individuals. Using published data of the other two seadragon species, we found that lineages of common seadragons have diverged relatively recently (< 0.63 Ma). Within common seadragons, we found pronounced genetic structure, falling into three major groups in the western, central, and eastern parts of the range. While populations across the Bassian Isthmus were divergent, there is also evidence for secondary contact since the passage opened. We found a strong cline of genetic diversity from the range center tapering symmetrically towards the range peripheries. Based on their genetic similarities, the poached individuals were inferred to have originated from around Albany in southwestern Australia.

**Conclusions:**

We conclude that common seadragons constitute a single species with strong geographic structure but coherence through gene flow. The low genetic diversity on the east and west coasts is concerning given that these areas are projected to face fast climate change. Our results suggest that in addition to their life history, geological events and demographic expansions have all played a role in shaping populations in the temperate south. These insights are an important step towards understanding the historical determinants of the diversity of species endemic to the Great Southern Reef.

**Supplementary Information:**

The online version contains supplementary material available at 10.1186/s12915-023-01628-9.

## Background

Species are the cornerstone of biology yet their definition remains challenging, not just because of various species concepts, but also because genome-wide assessments have demonstrated that the genome is often porous with gene flow across previously delineated species [1–5]. Genetic markers can be used to delimit the number of unique genetic lineages, both at the species- and population-level, which are the basic management units if conservation actions are necessary [6]. Many concepts consider species as populations that are connected by gene flow [7]. Yet, numerous examples show lineage divergence is often not a simple bifurcation caused by a cessation of gene flow, but can involve gene flow after initial split, secondary contact of previously isolated lineages, or even lineage fusion [8–11]. Not accounting for these processes runs the danger of over-splitting highly structured but demographically connected populations into “species” if not interpreted conservatively [12–14].

**Fig. 1:**
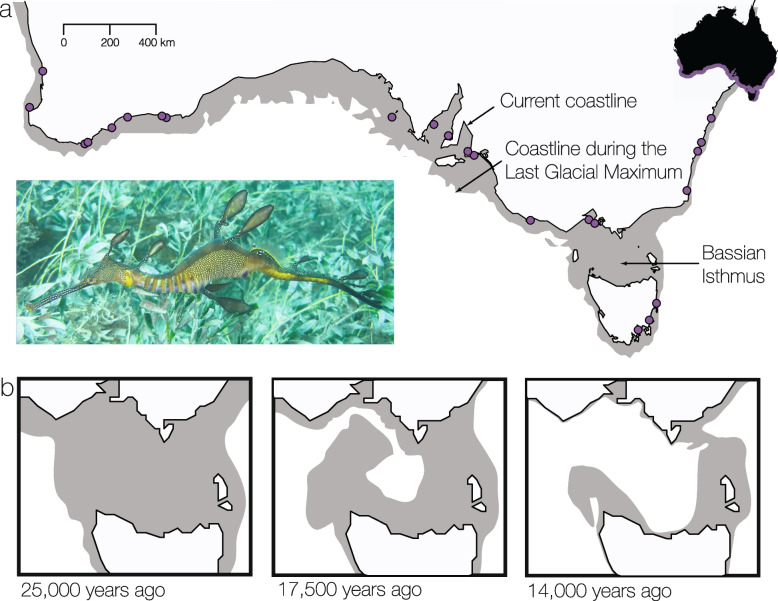
The range of common seadragons spans the southern Australian coast, including a known geogenetic break. **a** Sampling localities (purple dots) of *Phyllopteryx taeniolatus* along the entire known range (purple shade on map inset). The gray areas show the approximate location of the coastline during the Last Glacial Maximum, when sea levels were ca. 120 m lower than today. During sea-level lows, the Bassian Isthmus landbridge disconnected marine populations. The strait was long closed during the Penultimate Glacial Period (194–135 ka), followed by brief closures (76, 68–62, and 46 ka), and the most recent longer closure during the Last Glacial Maximum (43–14 ka) [18]. **b** Flooding sequence of the Bassian Isthmus with rising sea levels during the Last Glacial Maximum. Redrawn from [18]

Because some barriers to gene flow are temporary, their disappearance allows previously isolated populations to come into secondary contact. If gene flow is reinstated, previous genetic differentiation is eroded and the two lineages may fuse [15]. Secondary contact has been described in a number of terrestrial taxa, for example in populations that united after leaving glacial refugia [16, 17]. In the ocean, there are fewer clear examples where a previous barrier disappeared allowing for secondary contact. An example is Bass Strait in southeastern Australia, which contains a now-submerged land bridge connecting Tasmania and the mainland (Fig. 1a). This temporary barrier emerged due to the lowered sea levels during glacial periods [18]. After a long closure during the Penultimate Glacial Period (194 thousand years (ka) - 135 ka), the strait was mostly open until the Last Glacial Maximum [18]. When sea levels rose after the Last Glacial Maximum, the strait flooded from the west and fully opened around 14 ka ago (Fig. 1b). In many marine taxa, this separation caused genetic divergence on the east and the west of the Bassian Isthmus [19–21]. This divergence is maintained in genomes despite the possibility of secondary contact of the previously isolated populations over thousands of years. It is not always clear whether reproductive isolation is complete between lineages east and west of the former isthmus or whether gene flow has been reinstated since the opening.

The common seadragon (*Phyllopteryx taeniolatus* (Lacépéde, 1804), Syngnathidae) is distributed across Bass Strait and mitochondrial and nuclear analyses have left two interpretations of their divergence. While mitochondrial haplotypes found only shallow structure over Bass Strait (minimum uncorrected distance 0.12%) [22], a study using RADseq of the nuclear genome suggested deep divergence between populations east and west of the strait [23]. In the latter, the divergence between populations on the east coast and a population in Victoria was interpreted as distinct management units, and it was suggested that these units may be separate subspecies or species [23]. However, the methods employed in that study (F_ST_, Structure, DAPC) were not explicitly designed to uncover potential gene flow, leaving possible demographic connectivity across Bass Strait insufficiently addressed. Further, sampling was limited to the southeastern part of the range and therefore could only assess a subset of the genetic makeup of common seadragons.

Common seadragons have in fact a much broader distribution, spanning a total of 5500 km from the temperate west coast to the east coast extending north to Sydney and south to Tasmania (Fig. 1a). Among the three species of seadragons, also including leafy (*Phycodurus eques*) and ruby seadragons (*Phyllopteryx dewysea*) that are all endemic to southern Australia, common seadragons have the largest range. They inhabit shallow (usually < 30 m) kelp and seagrass beds [24] to which they are uniquely adapted with camouflaging appendages and color patterns. Common seadragons are thought to be poor dispersers because they swim slowly [25, 26] and lack a dispersive larval phase as the males brood the juveniles until hatching [27]. A range-wide assessment based on mitochondrial DNA suggested deeply diverged lineages between western Australia and the central and eastern parts (minimum uncorrected distance 1.3%) [22], but this needs validation with multiple unlinked genetic markers. Because the range of common seadragons spans the entire Great Southern Reef, a globally significant region that harbors high biodiversity with considerable endemicity [28, 29], a genomic assessment will be informative for other species of this important temperate reef system.

Range-wide sampling of common seadragons allows us to address three currently unknown issues that have direct conservation relevance. First, obtaining population characteristics for common seadragons across their entire range is critical to identify the number of distinct genetic units and to underpin their potential monitoring. Second, estimates of genetic diversity for populations across the range are useful as they are often seen as a proxy for the evolutionary potential and resilience to changes in their environment [30]. Third, range-wide sampling allows forensic investigation of the geographic origin of poached or illegally collected individuals whose source may be unknown [31–33]. Such approaches have so far found little application on ornamental fishes sold for the public and private aquarium trade, although they are traded in the millions with often little monitoring [34]. Common seadragons are extremely popular with visitors of public aquaria worldwide. All seadragons in aquaria come from the wild, although nowadays only few brooding males are taken and individuals raised from the eggs are exported [35]. Their capture requires permits due to the protection by the federal *Environment Protection and Biodiversity Conservation Act 1999*. Even though illegal capture does not seem to occur on large scales [35, 36], a black market may be motivated by the high prices that common seadragons sell at. Understanding the geographic source of illegally captured individuals is important to enact meaningful protection, which might use very different actions depending on the vulnerable location.

Here, we sequence genome-wide markers (Ultraconserved Elements, UCEs) for 198 individuals of common seadragons (*Phyllopteryx taeniolatus*) sampled along the entire range to investigate genetic structuring and genetic diversity. Because UCEs can be identified across divergent species (an advantage over most RADseq approaches), we were able to integrate the data with published UCE sequences of ruby seadragons and leafy seadragons to provide a time frame for inter- and intraspecific divergences. In order to assess the species status of common seadragons, we assess divergence and potential connectivity over Bass Strait. Lastly, we use the range-wide genetic framework to infer the geographic origin of two confiscated common seadragons.

## Results

We generated sequence data for a total of 198 *Phyllopteryx taeniolatus* spanning their known range (Fig. 1, Additional File 1: Table S1). Samples had on average 1.37 million read pairs (range 0.16–9.49 million reads, Additional File 2). After filtering (depth ≥ 10x, minor allele frequency > 0.05), 3643 SNPs with an average depth of 21.6x (range 5.48–56.40x) remained. We also included published UCE data for leafy and ruby seadragons for a total of 268 samples, resulting in 13,748 SNPs at an average depth of 22.5x (range 4.78–80.26x). We also obtained mitochondrial genomes for 155 samples of the three seadragon species (140 common seadragons, 14 leafy, 1 ruby) as “molecular by-catch” of the UCE target enrichment.

### Age of divergence between the common seadragon lineages

**Fig. 2:**
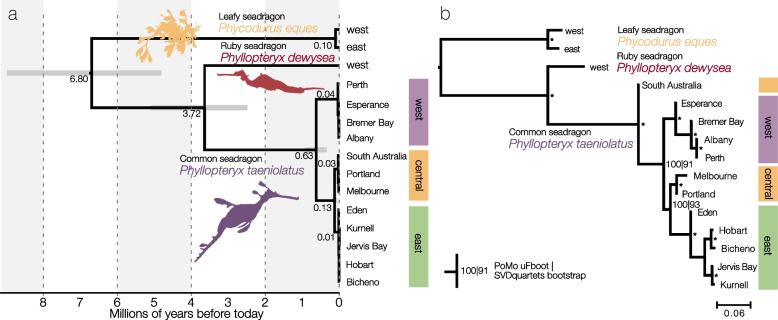
Relationships between seadragon species and within common seadragons. **a** Multi-individual species tree with dated divergences based on mitochondrial protein-coding genes using STARBEAST2 (155 individuals total, 14,075 base pairs). The ages of divergence among the lineages of common seadragons are very recent compared to the divergences among the seadragon species. **b** Multi-individual species tree of leafy, ruby, and common seadragons based on nuclear SNPs using PoMo (268 individuals, 13,748 SNPs). The topology from SVDquartets was identical (Additional File 1: Fig. S1); bootstrap support values from both analyses are annotated, with asterisks indicating full support in both analyses

In order to estimate a time frame of the divergences between the three seadragon species and between the lineages of common seadragons, we analyzed mitochondrial genomes using STARBEAST2. We performed divergence time estimates on the mitochondrial dataset because accepted molecular clocks for UCEs do not exist and no fossils are known for seadragons or their close relatives that could be used to inform calibrations. The constrained age of the most recent common ancestor of the three seadragon species was dated to a median of 6.80 Ma (95% HPD interval: 4.81–8.98 Ma, Fig. 2a). The divergence of ruby and common seadragons was inferred to a median of 3.72 Ma (95% HPD 2.48–5.09 Ma), which is broadly congruent with the estimate of 4.42 Ma of a phylogenomic study that interpolated ages based on distant fossil relatives [37]. The most recent ancestor of common seadragons was dated to 0.63 Ma (95% HPD 0.34–0.92). Individuals fell into three major groups corresponding to sampling sites in the west, in the central parts, and on the east coast. The eastern and central lineages were most closely related with a divergence time estimated to 0.13 Ma (95% HPD 0.07–0.19 Ma). Within the three main lineages, divergences were very recent (median < 0.01–0.04 Ma, 95% HPD 0.00–0.05).

Species tree analyses of the nuclear UCE data (SVDquartets, PoMo) obtained a similar topology (Fig. 2b, Additional File 1: Fig. S1). A main difference to the mitochondrial phylogeny was in the position of the South Australian population, which the nuclear data placed as the sister group to all other lineages, while the mitochondrial genomes supported it as the sister group to Melbourne and Portland (Fig. 2a). Another difference concerned the splitting sequence within the western group, with either Esperance (nuclear trees) or Perth (mitochondrial tree) being the sister group to the other western populations (Additional File 1: Fig. S2). In all species trees, Eden was placed as the sister group to all other east coast populations, albeit being in the center of the eastern coast.

### Population structure of common seadragons

**Fig. 3:**
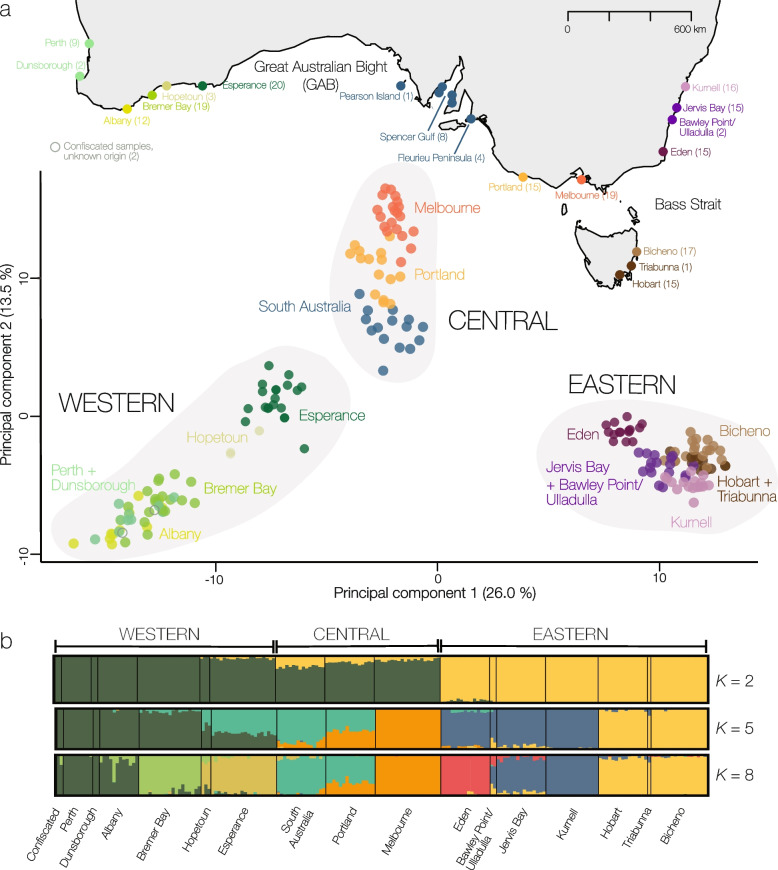
Population structure of common seadragons. **a** Principal component analysis (PCA) based on the data set without missing data (183 SNPs) showing the first two PCs. Individuals are represented as dots, colored by geographical origin reflecting the map above. **b** Individual genetic assignment as inferred by structure for *K* = 2, *K* = 5, and *K* = 8 based on the unlinked variants (961 SNPs)

The three main lineages of common seadragons (west, central, east) were also found in clustering analyses of individual samples. PCA showed a west–east orientation along the main axis (Fig. 3a, 26% of variation). Within the western group, the southwestern localities (Perth, Dunsborough, Albany, Bremer Bay) formed a broad assemblage, separate from individuals from Esperance. Individuals from Hopetoun, which is geographically located between Bremer Bay and Esperance, were found between the two clusters but closer to Esperance. The central group contained localities in South Australia, Portland, and Melbourne, which were oriented along the second PCA axis (13.5% of variation). On the east coast, localities from Sydney to Tasmania (Kurnell, Jervis Bay, Bawley Point/Ulladulla, Bicheno, Triabunna, Hobart) formed a tightly overlapping cluster. Eden formed a separate group from this cluster.

Individual assignment to genetic clusters showed increasing substructure at larger values of ancestral populations (*K*, Additional File 1: Fig. S4) rather than a single optimal value for *K*. Cross-entropy was minimized at *K* = 8 (Additional File 1: Fig. S3) and is therefore the highest value shown here. At *K* = 2, the samples separated into a western + central and an eastern group (Fig. 3b). The separation was however not complete because small proportions of the eastern ancestry component were present westward until Hopetoun. Conversely, Eden individuals had small proportions of the western + central ancestry component. The central group formed its own ancestry component at *K* = 3, Eden split at *K* = 4, and further substructure was detected in all groups at *K* > 5 (Additional File 1: Fig. S4). At *K* = 8, distinct ancestry components but usually with admixture with adjacent populations existed, three in the western group (Perth + Dunsborough + Albany, Bremer Bay, Hopetoun + Esperance), two in the central group (South Australia + Portland, Melbourne), and three in the eastern group (Eden, Bawley Point/Ulladulla + Jervis Bay + Kurnell, Hobart + Triabunna + Bicheno).

Most sampling sites differed significantly in their allele frequencies (Additional File 1: Fig. S5). Pairwise differentiation was lowest between the adjacent localities (Esperance versus Hopetoun, F_ST_ = 0.06; Jervis Bay versus Kurnell, F_ST_ = 0.08), and highest between the extremes of the range (Perth versus Kurnell, F_ST_ = 0.73). The western and the central groups were significantly differentiated, albeit moderately considering the large geographic distance between the closest sites (F_ST_ = 0.15 over ca. 1300 km separating Esperance and Pearson Island). Between the central and the eastern group, minimal differentiation was higher (F_ST_ ≥ 0.41), yet differentiation was generally also high between adjacent localities of the east coast, for example Eden versus Kurnell (F_ST_ = 0.41).

### Secondary contact and gene flow

**Fig. 4:**
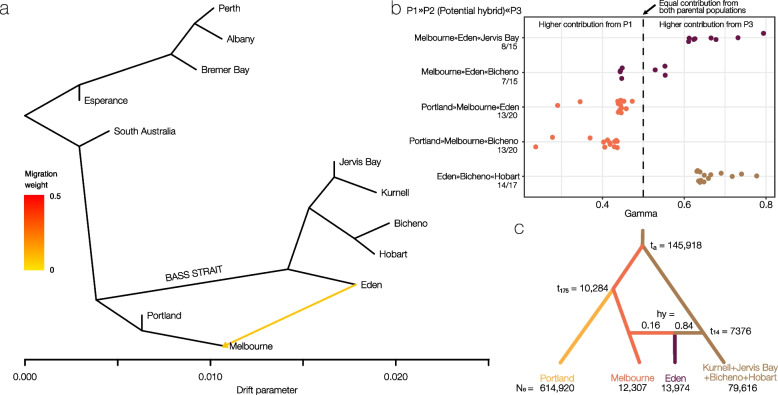
Evidence for secondary contact over Bass Strait. **a** TreeMix analysis on the unlinked variants (961 SNPs) allowing for one migration event, showing admixture across Bass Strait from Eden to Melbourne (yellow arrow). The analysis is based on allele frequencies of populations using all individuals. Branch lengths are proportional to the amount of inferred genetic drift. **b** Results from HyDe analysis showing the strongest signal of introgression between Melbourne and Eden. Only comparisons which were statistically significant after BH correction are shown. For each putative hybrid individual (P2), a Gamma of 0.5 is expected for hybrids between two parental populations (P1, P3), while values closer to 0 indicate a higher contribution is from P1, and values closer to 1 arise from a higher contribution of P3. Fractions under population labels indicate how many individuals of the potential hybrid population showed a significant signal of introgression. **c** Estimated parameters for the favored scenario using DIYABC Random Forest, supporting secondary contact across Bass Strait. Shown are mean values (quantiles in Additional File 1: Table S2)

In order to account for possible gene flow, we used TreeMix to infer population splits and superimpose potential migration events. TreeMix recovered the same topology as the other methods based on the nuclear data (Fig. 2b), but inferred a migration event from Eden to Melbourne across the former Bassian Isthmus on top of this species tree (Fig. 4a). Evidence for gene flow was also supported by the *D* and Gamma statistics. For the *D* statistics, all statistically significant results supported gene flow between Melbourne and Eden, with different combinations of P1, P2, and P3 populations (Table 1, *D* = 0.13–0.21, *z*-score > 3.39, Benjamini-Hochberg (BH) adjusted *p* < 0.05). The same population trios involved in introgression were identified by HyDe, but not all individuals in the potential hybrid population showed statistically significant deviation from expected site frequencies (Fig. 4b, 46–82% of population of hybrid origin, BH adjusted *p* < 0.05).

**Table 1:** *D* statistics calculated among populations of common seadragons

P1	P2	P3	ABBA	BABA	D	f4-ratio	*Z*-score	*p*	*p* adj
Hobart	Eden	Melbourne	72.688	47.532	0.209	0.152	4.472	7.75E − 06	1.71E − 03
Portland	Melbourne	Eden	114.332	87.309	0.134	0.097	4.244	2.20E − 05	2.42E − 03
Kurnell	Eden	Melbourne	78.502	53.009	0.193	0.147	3.989	6.65E − 05	4.87E − 03
Jervis Bay	Eden	Melbourne	78.363	56.56	0.161	0.124	3.44	5.82E − 04	3.20E − 02

Out of all tested trios of common seadragon populations (220 comparisons), 4 comparisons were statistically significant after BH correction (*p* adj. < 0.05); other comparisons are in Additional File 1: Fig. S6. Positive *D* statistics represent an excess of sites supporting ABBA versus BABA pattern, indicating potential introgression between taxa P2 and P3. Ruby and leafy seadragons were used as the outgroup

We used a supervised machine learning algorithm (DIYABC Random Forest) to select among models of divergence and potential secondary contact across Bass Strait. The selected scenario was significantly favored over the alternatives (model 2 votes = 1370, model 1 votes = 507, model 3 votes = 123, Additional File 1: Fig. S7). Under this model, populations were diverging allopatrically for a period, followed by a more recent admixture event between Melbourne and the remaining east coast populations (Jervis Bay, Kurnell, Hobart, Bicheno), which resulted in the formation of the Eden population (Fig. 4c). The divergence of the Victorian and east coast populations was dated to a median of 145,918 years ago (95% quantile: 71,941–190,782, Additional File 1: Table S2), similar to the mitochondrial divergence dates (Fig. 2a). The Portland and Melbourne populations were estimated to have diverged 10,284 years ago (95% quantile: 3321–16,884). The admixture was inferred to have occurred 7376 years ago (95% quantile: 1731–13,015), with an admixture rate of 0.16 from Melbourne into Eden (0.06–0.53).

### Genetic diversity

**Fig. 5:**
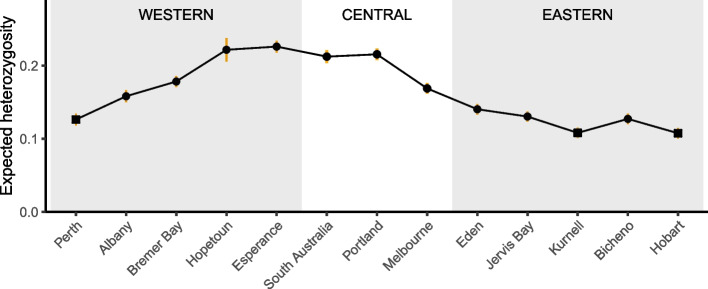
Genetic diversity of common seadragons declines away from the range center. Populations are arranged from west to east, and dots represent population-level expected heterozygosity (H_e_, 3643 SNPs), with line ranges indicating 95% confidence intervals around the mean. The squares highlight populations close to the range limits; Perth at the northwestern range edge, Kurnell at the northeastern edge, and Hobart at the southeastern range limit

Genetic diversity (H_o_ and H_e_, Table 2) showed a clear geographic pattern being highest in the center of the range, particularly in Hopetoun, Esperance, South Australia, and Portland (H_e_ = 0.21–0.23), and declining both east- and westward (Fig. 5). The eastern group had low genetic diversity throughout (H_e_ = 0.11–0.14). The three populations at the range edges all had the lowest values of genetic diversity (Fig. 5, H_e_ = 0.11–0.13).

**Table 2:** Genetic diversity estimates for populations of common seadragons

Population	*N*	*H*_*o*_	*H*_*e*_
Perth	11	0.14	0.13
Albany	14	0.17	0.16
Bremer Bay	19	0.19	0.18
Hopetoun	3	0.23	0.22
Esperance	20	0.27	0.23
South Australia	15	0.21	0.21
Portland	15	0.24	0.22
Melbourne	20	0.18	0.17
Eden	15	0.16	0.14
Jervis	17	0.14	0.13
Kurnell	16	0.12	0.11
Bicheno	17	0.14	0.13
Hobart	16	0.12	0.11
Total	198	0.20	0.17

*N* number of samples per population, *H*_*o*_ observed heterozygosity, *H*_*e*_ expected heterozygosity

### Origin of confiscated samples

Our range-wide sampling for common seadragon populations, sequenced for hundreds of unlinked SNPs across their genome, provided the genetic baseline for tracing the origin of traded seadragons. We used this framework to genetically infer the most likely origin of two samples of common seadragon that were seized after illegal export. Population assignment using likelihood ratios assigned the individuals to the Albany reference population with a much higher likelihood than other localities (Additional File 1: Table S3). Likelihoods of origin from other locations decreased with distance from Albany.

**Fig. 6:**
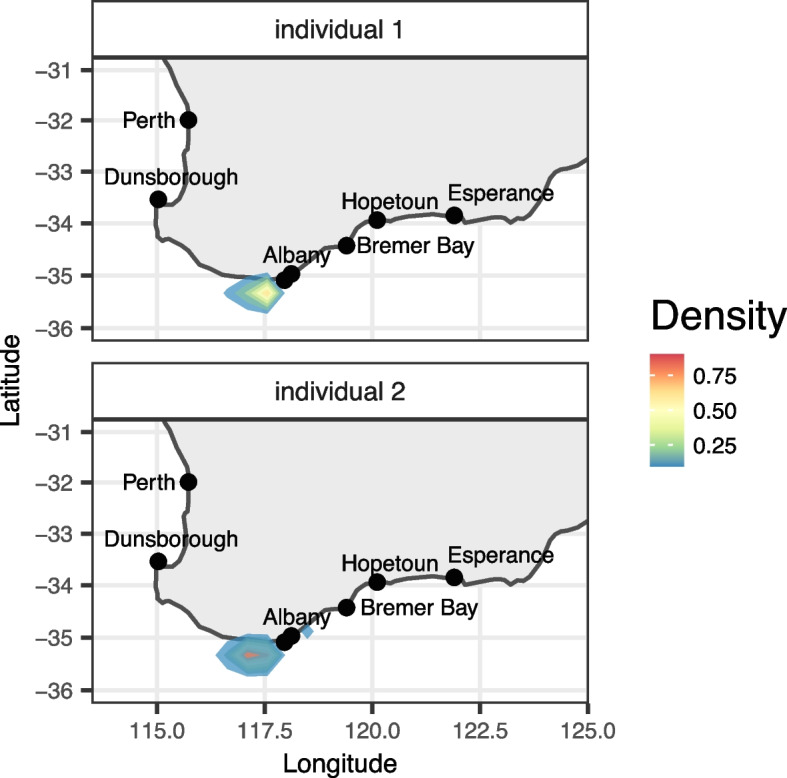
Estimated location of the two confiscated individuals of common seadragons in southwestern Australia. The inference employed SCAT based on the dataset without missing data across the entire range (183 SNPs). Colors show the density of inferred geographic coordinates for the sample origin from 10 independent MCMC runs (5000 post-burnin samples) with warmer colors indicating regions of frequent inference

To allow for the possibility that the samples originated in a region other than the reference populations sampled here, we used SCAT [32] to infer likely geographic origins of the two samples. The highest density of inferred geographic origins for both samples was around Albany, but supported a highest probability slightly west of it (Fig. 6). The highest density for individual 1 was off West Cape Howe National Park (around 117.560 E 35.331 S). The highest density for individual 2 was more diffuse and was located off Owingup Nature Reserve (around 117.094 E 35.334 S). These results strongly indicate a southwestern origin of these two individuals, with the most likely origin from around Albany.

## Discussion

We used UCEs and mitochondrial genomes to study the range-wide genetic structure and genetic diversity of the common seadragon *P. taeniolatus*. The wide distribution of this species allowed us to investigate its genetic makeup across the entire temperate Australian coast and specifically in relation to the geologic history. The data demonstrated (1) strong genetic structuring in three major groups with substructure in each group, but evidence for genetic cohesion among the groups, (2) a significant genetic disjunction across the Bass Strait with evidence for secondary contact, and (3) a cline in genetic diversity from the central regions and the eastern and western parts of the range and (4) allowed us to infer that two illegally exported samples likely originated from around Albany in Western Australia.

### Common seadragons are strongly geographically structured yet a single species

Our analyses of mitochondrial genomes and genome-wide SNPs from UCEs showed strong geographic structuring into three main groups, in the west, central, and east coast of Australia, which each showed additional population structure among sampling sites (Fig. 3, Additional File 1: Fig. S5). Given that common seadragons have a generally poor dispersal ability [25], this strong geographic structuring is not surprising and is in line with previous mitochondrial data [22]. 

Despite the strong geographic structure, our data clearly shows shared genetic variants between the three main groups and adjacent populations, indicating genetic connectivity between neighboring populations. When viewed in relation to the age of divergence of common seadragons and the other seadragon species, the intraspecific divergences among common seadragons lineages were much more recent (Fig. 2a). The divergence over the former Bass Strait was here shown not to be the oldest split of common seadragons, but is only one of the genetic subdivisions present across the range. This implies that while there is substantial population structure in common seadragons, these do not constitute old and sustained divergences as among common, leafy, and ruby seadragons.

#### Isolation and secondary contact over Bass Strait

The genetic divergence in the southeast across Bass Strait separating the central clade from the eastern group was pronounced across all analyses. As in other marine taxa, this genetic break of common seadragons was likely caused by repeated isolation on either side of Bass Strait during sea level lows [19, 21]. In common seadragons, the onset of isolation appears to predate the last closure of the Bassian Isthmus because the divergence across Bass Strait was here dated to ca. 130 ka, shortly after the Penultimate Glacial Period (194–135 ka). This is evidence that previous glacial periods, not just the most recent one, have shaped these populations.

Despite this main pattern of isolation across the genome, we also found substantial evidence for secondary contact between the previously isolated lineages east and west of Bass Strait, estimating that 16–21% of genetic variation is shared between Eden and Melbourne. Secondary contact may also explain the atypical position of the Eden population in species tree analyses, where it was the sister group to all other east coast populations, albeit being geographically located in the center of the eastern populations. The D statistics further indicated that the exchanged genetic material has spread along the east coast into all sampled populations, although these results were not statistically significant after correcting for multiple testing. We suggest that greater genetic marker density and sampling around the secondary contact zone could identify additional admixed populations. Overall, these analyses provide evidence for reinstated genetic exchange across Bass Strait.

The sustained imprints of a historical landbridge that disappeared 14,000 years ago suggest that rates of admixture are low. It is possible that there is some pre- or postzygotic incompatibility that may slow down genetic exchange [38], but there is no data that would allow us to assess this in common seadragons. It may be more likely that the sustained genetic differences are simply a consequence of the low dispersal ability of this brooding, slow-swimming fish. Lastly, the convergence of two boundary currents in the area [20, 39, 40] or gaps in rocky habitat in the eastern Bass Strait may also slow down mixing [21, 41].

Our results stand in contrast to a previous study that concluded that there is no evidence of gene flow and suggested raising a subspecies [23]. The discrepancy in conclusions is likely attributable to the methods employed, not the genetic markers used. Where our sampling overlapped in populations of the east coast and one population of the central group, we observed similar levels of population differentiation (F_ST_) and genetic diversity (H_o_, H_e_) estimated from the UCE loci used here and from the RADseq loci used in [23] (Additional File 1: Fig. S8). This is despite sampling different individuals and employing different bioinformatic procedures, which can complicate comparisons between these two different genetic markers [42]. On the other hand, the methods used in the previous study [23] (F_ST_, DAPC, and structure analysis [23]) are not directly suited for detecting gene flow [43], while we tested for potential admixture with methods developed for this task (TreeMix, *D* statistics, Gamma statistics, model testing). Models that only consider lineage divergence are now known to oversplit population-level differentiation [13, 14]. Our results illustrate the potential of genomic data for detecting both divergence and secondary contact in order to gain a complete picture of lineage delimitation.

#### Divergence between the western and the central group

A notable difference in the inferred patterns between mitochondrial genomes and genome-wide nuclear SNPs concerns the degree of divergence between the western and the central group. In agreement with a previous study based on two mitochondrial fragments [22], we found a sizable divergence in mitochondrial genomes. The nuclear SNPs on the other hand did not support strong divergence, with some admixture components being shared between South Australia and Esperance and relatively low genetic differentiation considering the large geographic distance between the closest sampling sites. The disagreement between mitochondrial and nuclear patterns may be explained by the lower effective population size of the maternally inherited mitochondrial genome, which may result in faster sorting of lineages after a divergence event [44, 45].

The low divergence in nuclear DNA could be evidence of high gene flow but this is not expected given the considerable geographic distance and the low dispersal of common seadragons. Another explanation is a recent population divergence. It is possible that the ancestral populations were geographically closer during sea-level lows and then separated geographically when the coastlines shifted with rising sea levels. This is similar to leafy seadragons, for which genetic and geological modeling suggested that localities around South Australia and the central coast may have served as refugia from which recolonization occurred after sea-level rise [46]. Such a scenario could also explain the position of the South Australian population as the sister group to all other populations in species-tree analyses of the nuclear data (Fig. 2b). Without samples from this largely inaccessible intermediate area, it may remain speculation if the western and central groups are truly isolated or are continuously connected.

### Cline in genetic diversity towards the range edges

We found a pronounced cline in genetic diversity with high diversity in central locations and tapering diversity going both east- and westward. The pattern is remarkably similar to the leafy seadragon, which shows a cline from the central regions westward but is not distributed east of South Australia [46]. The pattern in the more broadly distributed common seadragon now shows that this tapering diversity is mirrored both east- and westward. Similar broad sampling across the entire south is rare but habitat-forming seagrasses and macroalgae may show the same pattern. The seagrass *Posidonia australis* also has lower genetic diversity through New South Wales compared to Victorian and South Australian populations [39], and genetic diversity decreases towards the range edge in New South Wales [47]. The kelp *Ecklonia radiata* shows a strong decline in genetic diversity northward on Australia’s west coast [48]. Additional species should be characterized across the Great Southern Reef to investigate whether this tapering diversity pattern is a shared feature.

Two explanations have been proposed to explain the often reduced genetic diversity at range edges. The center-periphery hypothesis states that genetic diversity is highest in the center of a species’ range where environmental conditions are ideal, while the range edges have suitable conditions [49]. The rear-leading edge hypothesis attributes the spatial patterns of genetic diversity to past climatic changes, which caused colonization from stable refugia to peripheral areas resulting in a loss of genetic diversity [16, 50–52]. In common seadragons, we can envision that both processes may have been at play. The central region has a broad shelf, which may harbor more suitable habitat than the peripheral regions [46]. Repeated founder dispersal east- and westward may have produced the observed slope of genetic diversity. The peripheral populations of common seadragons are located on the eastern and western coasts, where temperature gradients are steeper than in the central parts of the range. In these areas, historical changes in sea surface temperature [53] may have repeatedly moved range limits up and down the coast, resulting in lowered genetic diversity.

### Origin of illegally captured samples inferred to southwestern Australia

The genetic assignment of the two confiscated individuals of common seadragons to populations in southwestern Australia was unambiguously supported by all analyses, with the highest support for an origin west of Albany. The coast west of Albany encompasses several remote National Parks, with much of the coastline between Albany and westward to the Capes being inaccessible and exposed to inclement weather. These types of areas are challenging for ongoing surveillance.

The forensic approach employed here could also be useful for other ornamental fish species. Currently, forensic approaches on aquarium fishes are used mostly for species identification employing mitochondrial barcodes [54]. However, mitochondrial barcodes or even mitochondrial genomes usually offer limited resolution to identify lineages within a species at the geographic resolution needed for forensic inference [33]. Multiple genetic loci across the genome offer the advantage of increased resolution to infer geographic origin and the use of probabilistic methods to interpolate between reference populations [32]. Our range-wide sampling of reference populations and multiple genetic loci provided the scaffold to infer the most likely geographic source of these samples. It is possible that even more fine-scale sampling of reference populations would improve the triangulation of illegally captured individuals. Further, whole genomic resequencing would provide more diagnostic variants for particular geographic locations, which could add power to narrow down geographic origin.

### Conservation implications

The question of whether the east coast common seadragons are demographically independent from the central coast has practical implications if conservation actions become necessary. Separate management of the east coast and the central populations, as suggested before [23], may have unintended consequences by interrupting gene flow across Bass Strait. Our analysis suggests that low levels of gene flow occur across Bass Strait and management actions should jointly assure that these populations can remain in contact through connecting habitat. This is not to say that the east coast populations should not be monitored carefully; their low genetic diversity is reason for concern, particularly in the light of projected rapid climate change in the area [55]. The same is true on the west coast, where the already low genetic diversity of the Perth population raises similar issues. We currently do not know whether marine heat waves in recent years have resulted in further losses of genetic diversity compared to the baseline provided here. In kelps, significant demographic changes and loss of genetic diversity have been attributed to the effects of heat waves [56, 57]. Finally, fine-scale genetic information may assist in investigating cases of illegal capture and exportation of these iconic temperate fish.

## Conclusions

Our genomic assessment of common seadragons provided a detailed view of their range-wide structuring, connectivity, and genetic diversity. These insights will inform conservation prioritization and may be used to address illegal wildlife trade. They also emphasize that species delimitation needs to consider gene flow. The results indicate a strong geological legacy in the genomes of common seadragons, with lineages shaped by vicariance and reunion, and their genetic diversity tapering towards the range edges. The historical geology of the temperate Australian coast likely impacted most shallow-water species, although the imprints on the genome may vary depending on the dispersal rate. In order to understand the history of inhabitants of the Great Southern Reef, we therefore need to consider life history and geological history driving divergence, secondary contact, and demographic fluctuations.

## Methods

### Sampling

A total of 198 individuals of common seadragons (*P. taeniolatus*) were sampled across the species’ range (Fig. 3, Additional file 1: Table S1), including one sample from a previous study using UCEs [58, 59]. In order to assess the degree of divergence between lineages of common seadragons in relation to the other members of the seadragon clade, we integrated data from previous studies using UCE loci including two samples of ruby seadragon (*Phyllopteryx dewysea*), one from the holotype and one beach-washed individual [37, 60] and 68 samples of leafy seadragon (*Phycodurus eques*) [46, 61].

Common seadragon samples came from a previous study (which sequenced only mitochondrial DNA fragments [22]), as well as one sample from a study that used the same UCE markers for phylogenetic purposes [58], and additional tissues from previously unsampled individuals (*N* = 26). Of these, tissue clips were taken from wild fish (*N* = 14) and others came from preserved samples (*N* = 10), specifically from the Western Australian Museum Perth (*N* = 4), the South Australian Museum Adelaide (*N* = 1), the Australian National Fish Collection Hobart (*N* = 2), our own tissue collection (*N* = 1, Hobart), and from individuals held at Birch Aquarium at Scripps, which were raised from a brood collected in Melbourne (*N* = 2). Samples of common seadragons collected illegally (*N* = 2) were provisioned by the Western Australian Museum Perth, donated by the Department of Fisheries, Western Australia.

For analyses that require population assignment, samples were grouped by sampling locality as these were genetically identifiable (F_ST_ > 0.08, see Results; Additional File 1: Fig. S5). Localities with a low number of samples (*N* < 5) were analyzed with the population they clustered most closely in individual-level assignments (see Results): Dunsborough (*N* = 2) was analyzed with Perth, Hopetoun (*N* = 3) with Esperance, samples from localities in South Australia were analyzed together (Pearson Island (*N* = 1), Spencer Gulf (4 sites with *N* = 1–4), Fleurieu Peninsula (3 sites with *N* = 1–2), Bawley Point/Ulladulla (*N* = 2) with Jervis Bay, Triabunna (*N* = 1) with Hobart.

### Library preparation, UCE target enrichment and sequencing

DNA was extracted from dermal or muscle tissue (dried or stored in ethanol) using the DNeasy Blood & Tissue kit (Qiagen). We quantified DNA using a Qubit fluorometer (Life Technologies). DNA was sheared by sonication with a Bioruptor Standard (Diagenode) into fragments of an average size of 400–700 bp. Genomic DNA libraries were prepared using a commercial kit following the manufacturer’s instructions (KAPA Biosystems LTP Library Preparation Kit or KAPA Hyper Prep Kit) with an input of 60–1200 ng DNA. DNA was cleaned using a SPRI bead substitute [62]. Individual libraries were indexed with a single- [63] or dual-sequence barcodes [64]. For target enrichment, 8 libraries were pooled at equimolar ratios (62.5 ng each). Enrichment targeted 1314 UCE loci [65] were enriched with commercially synthesized RNA probes (UCE Acanthomorph 1Kv1, MyBaits, Daicel Arbor Biosciences) following the protocol recommended by the manufacturer (versions v2 and v3).

Samples were sequenced using two runs of MiSeq (Illumina, Inc.) with 600 cycle (= 300 bp paired end, PE) v3 chemistry and with five runs of 500 cycle (= 250 bp PE) v2 chemistry at the UCSD Stem Cell Genomics Core and one run of 500 cycle v2 chemistry at the UCLA Genomics Core. We also shared three lanes of HiSeq2500 (Illumina, Inc.) in rapid run mode (100 bp PE), and one lane of the HiSeq4000 (Illumina, Inc.) (100 bp PE) at the UCSD IGM Genomics Center.

### Bioinformatic processing

Raw reads were cleaned from adapter contamination and low-quality bases with trimmomatic v.0.39 [66] using illumiprocessor v.2.0.2 [67]. We produced a reference assembly of UCE loci and their flanking regions for one common seadragon (Portsea, individual code PSA) using scripts implemented in phyluce v.1.5 [68]. We assembled contigs with velvet v.1.2.10 [69] with k-mers ranging from 25 to 75 in increments of 10. Among these contigs, we selected the longest contig for each UCE locus as the reference. Sequence reads of each sample were mapped against the reference with bwa v.0.7.17 mem [70] and processed with samtools v.1.9 [71]. The following steps were performed with tools implemented in gatk v.4.1.4.0 [72, 73]. SortSam was used to sort bam files by coordinates, MarkDuplicates was used to filter PCR duplicates, read groups were added with AddOrReplaceReadGroups, and BAM files from individuals that were sequenced on multiple sequence runs were combined with MergeSamFiles. Sequencing, mapping, and deduplication statistics for runs and samples are given in Additional File 2.

We created two sample sets, one including all samples of common, leafy, and ruby seadragons (*N* = 268), and the other of common seadragons only (*N* = 198). Both sample sets were processed and filtered in the same manner. SNPs for each sample were called with HaplotypeCaller in GVCF mode, combined with CombineGVCFs, and genotyped using GenotypeGVCFs. Variants were filtered for quality using VariantFiltration and only biallelic SNPs were retained. vcftools v.0.1.17 [74] was used to include only genotypes with ≥ 10 × depth of coverage in each individual and with a minor allele frequency of ≥ 0.05. Across the 268 samples, a total of 13,748 SNPs were identified. Across the 198 common seadragons, 3643 SNPs were identified. The latter dataset was further filtered to meet the requirements of downstream analyses, including a dataset without missing data (183 SNPs) and an unlinked dataset with one SNP per locus (986 SNPs).

### Assembly of mitochondrial genomes

Due to the natural abundance of mitochondrial genomes in cells, sequences originating from the mitochondrial genome can be included as “by-catch” of targeted capture of UCEs [75], particularly with earlier versions of the targeted capture protocol (v2). In order to extract potential mitochondrial sequence reads from the trimmed sequence data, we used mirabait v.4.0.2 [76] against mitochondrial genomes of leafy and common seadragons to identify potential mitochondrial reads. The reads were assembled and protein-coding genes, tRNAs, and ribosomal RNAs were annotated using MitoFinder v.1.3 [77]. Assemblies did not always circularize, likely because of the difficulty in assembling the control region but were otherwise complete. A total of 155 samples (140 common seadragons, 14 leafy seadragons, 1 ruby seadragon) produced almost complete mitochondrial genomes, which we defined as single contigs of > 16,000 bp length with annotations for all 13 protein-coding genes and the two rRNAs.

### Relationships and divergence times to other seadragon species

To investigate phylogenetic relationships among the three seadragon species and among common seadragons using species tree approaches, we used UCE sequence data and mitochondrial genomes in multispecies coalescent frameworks. The “species” estimated with multispecies coalescent methods do not necessarily represent taxonomic species but can be any group of individuals that have diverged through restricted gene flow [14, 78]. In order to estimate divergence times between seadragon lineages, a time calibration is needed. Because there are no known seadragon fossils that could be used, nor an established molecular clock for UCE loci, we used mitochondrial genomes for this analysis. We used a molecular rate of 0.01359655 (SD = 0.3) estimated for Pomacentridae [79, 80] in combination with a wide normal prior on the root based on a previously inferred age for the divergence of leafy and common seadragons (mean 6.94 Ma, sigma 2.4 for a 95% HPD 2.99–10.9 Ma, [37]). Sequences for each gene were aligned using MAFFT v7.130b [81]. We used IQTREE v.2.13 [82] to merge similar partitions, resulting in three partitions. For Bayesian calibration of the species tree, we employed STARBEAST2 v.0.15.13 [83], which allows integrating multiple individuals for each population. Because the mitochondrial genome is a single, maternally inherited locus, we set ploidy to 0.5, employed one uncorrelated, lognormal clock model and one Yule tree model, while allowing estimation of different substitution models for the three partitions using bModelTest v.1.2.1 [84]. Two independent runs were conducted using 10 million generations, with trees and log files sampled every 5000 generations. Assessment of convergence and tree annotation was done in Tracer v.1.7.2 [85].

For the nuclear data, we used a polymorphism-aware (PoMo) model, which reconstructs the evolution of lineages considering both changes due to mutations and changes in frequency of alleles for example due to genetic drift [86, 87]. The PoMo approach implemented in IQTREE uses frequency data per lineage to estimate lineage topology and branch lengths as number of mutations and as frequency shifts per site [88]. Because PoMo requires invariant sites in addition to the variants, we placed SNPs onto the reference loci (891,858 sites total), concatenated the alignment using AMAS [89], and prepared for analysis using FastaToCounts.py (https://pypi.org/project/cflib-pomo/). We inferred suitable models incorporating a parameter for polymorphisms (+ P) using ModelFinder [90] and assessed support with 1000 ultrafast bootstrap replicates [91]. We used the same dataset with SVDquartets [92] in PAUP v.4.0a168 [93], which reconstructs a species tree based on quartet trees under the multispecies coalescent model using unlinked variants [94]. A total of 50,000,000 quartets were sampled (39% of distinct quartets) and support was estimated using 100 bootstrap replicates.

### Population structure and genetic diversity of common seadragons

In order to elucidate population structure within common seadragons (198 samples), a principal component analysis (PCA) was performed on the dataset without missing data across all common seadragons with the ade4 R package [95]. We used Admixture v.1.3.0 [96] using the unlinked dataset. We tested for 1–15 clusters (*K*), repeated each 20 times, and summarized repeated runs on the clumpak web server [97].

We calculated pairwise differentiation between populations as F_ST_ in GenoDive v.3.06 [98] with 1000 permutations to assess statistical significance using all SNPs. Populations were grouped as in the species tree analysis. Genetic diversity was calculated using all SNPs with GenoDive for each population as observed and expected heterozygosity.

### Analysis of gene flow and admixture

Because the above species tree analyses cannot account for the possibility of gene flow across lineages, we used TreeMix v.1.13 [99] to infer the split history of lineages and to infer possible migration events. TreeMix uses allele frequencies, which were calculated from the unlinked dataset for each sampling locality.

In order to test for gene flow between the populations while accounting for the possibility of incomplete lineage sorting, we calculated ABBA-BABA or D statistics, which express the frequency of sites that are discordant with the species tree [100, 101]. Samples of leafy and ruby seadragon were used as outgroups, and common seadragon populations were tested in all combinations of population trios serving as populations P1, P2, and P3. Based on the full dataset (*N* = 268, 13,748 SNPs), we calculated the conservative *D*_min_ statistic with Dtrios of DSuite [102], i.e., the minimum *D* statistic for each trio of populations irrespective of the relationship between them [102]. Support was assessed with 100 jackknife blocks. Comparisons were corrected for multiple testing using the Benjamini-Hochberg (BH, [103]) method in R.

To account for the possibility that not all individuals of a population experienced the same degree of gene flow, we employed HyDe [104], which estimates deviations of site patterns for individuals of each target population. The Gamma statistic indicates the genetic contribution of the two parental populations for an individual’s genotype. As above, we tested all trios of populations, with leafy and ruby seadragon samples as the outgroup and correcting the resulting *p* values for multiple testing.

To test support for different divergence scenarios across Bass Strait and to estimate population parameters, we used DIYABC Random Forest v.1.0 [105]. This method uses bootstrapped decision trees (creating a “forest”) to perform classification using a set of predictor variables, which are the summary statistics obtained from genetic data. Random Forest classification requires fewer simulations and is not dependent on preliminary selection of relevant summary statistics, which can have a major influence on traditional ABC analyses [106]. We identified the most suitable model among three competing scenarios (Additional File 1: Fig. S7): (1) a strict isolation model without admixture; (2) a model of secondary contact, in which the eastern and central populations diverged in allopatry, after which admixture gave rise to the Eden population; and (3) a model of secondary contact, in which admixture gave rise to both Eden and Melbourne populations. We then estimated population parameters under the chosen model. From the unlinked SNP dataset, we filtered sites missing in more than half of the individuals and sites that were monomorphic across the four populations, leaving 539 SNPs. We simulated a training set of 100,000 data sets and calculated summary statistics for observed and simulated data to train the model. We used five noise variables and generated 2000 Random Forest trees to select the most likely scenario and estimated parameters using 10,000 out-of-bag testing samples. Prior values were drawn from uniform distributions (Additional File 1: Table S2). Lacking biological information, we set broad prior distributions for effective population sizes (100–1,000,000) and for the admixture rate (i.e., the proportion of genes of a source population entering the admixed population, 0.01–0.99). The prior for the age of the divergence of Portland and Melbourne (17,500–0 years) incorporated the fact that the Melbourne population could have only been established after the flooding of the former Bassian Isthmus because it was formerly on dry land (Fig. 1b). Gene flow across Bass Strait was allowed from 14 ka onwards. For other split events, we lacked geological information to inform prior distributions, and we therefore used broad priors with a maximum age on the beginning of the penultimate glaciation 195 ka. These priors are broad enough to include the 95% HPD intervals estimated using the mitochondrial genomes (Fig. 2a).

### Origin of confiscated samples

In order to infer the geographic origin of two confiscated individuals of unknown origin, we used a population assignment test implemented in GenoDive that calculates the likelihood that the individual’s genotype is found in a reference population based on allele frequencies [107]. Analyses were run with 100 permutations to assess the significance and a significance threshold of 0.002 as suggested [107]. To address the possibility that the confiscated samples came from another locality than the reference populations sampled here, we used SCAT v.3 (https://github.com/stephens999/scat) to interpolate the origin of individuals on a geographic grid. SCAT estimates smoothed maps of allele frequency variation based on reference populations and infers a probability distribution of the geographic source of each confiscated sample [32]. We defined a boundary file capturing the range of common seadragons (drawn with https://www.keene.edu/campus/maps/tool/). We ran 10 replicate analyses from different starting seeds with 1000 MCMC iterations each, of which half were discarded as burnin. All post-burnin inferred geographic coordinates from the replicate runs were combined and plotted using a 2D kernel density estimation.

## Supplementary Information

**Additional file 1: Fig. S1.** Multi-individual species trees for 268 individuals of leafy, ruby and common seadragons based on 891,858 base pairs including 13,748 SNPs. **Fig. S2.** Phylogenetic relationships based on mitochondrial genomesof 155 individuals of leafy, ruby and common seadragons. **Fig. S3.** Cross entropy values for 20 replicates of Admixture analyses, each runs for 1–15 ancestral populations. **Fig. S4.** Results of individual clustering of 198 individuals with Admixture. **Fig. S5.** Pairwise FST comparisons between populations. **Fig. S6.** Estimates of *D* statistics between pairs of populations. **Fig. S7.** Overview of the scenarios tested in DIYABC Random Forest. **Fig. S8.** Comparison of metrics of genetic differentiation and genetic diversity between the present study and the previous study by Klanten et al. 2020. **Table S1.** Sampling information for the 198 individuals of common seadragon. **Table S2**. Priors for the DIYABC Random Forest analysis. **Table S3**. Assignment of two confiscated samples based on population assignment in GenoDive using likelihood ratios.

**Additional file 2:** Sequencing statistics, mapping and deduplication statistics and SRA accessions for each sequencing run, each sample. Genbank accessions for the mitochondrial genomes.

## Data Availability

All raw sequence reads are deposited in the SRA under BioProject PRJNA895416 [108]. Mitochondrial assemblies are deposited in the NCBI Nucleotide Database under accession numbers OQ942702—OQ942856. Sample metadata are stored in the SRA and GenBank accessions, in addition to Additional File 1: Table S1. Other data files (raw and filtered VCF files, input and output files) and R scripts to reproduce the results are available from Figshare https://doi.org/10.6084/m9.figshare.21610362 [109]. This study used published raw sequence data from BioProjects PRJNA378844 [59], PRJNA734786 [60], and PRJNA624364 [61].

## Abbreviations

BH: Benjamini-Hochberg correction for multiple testing
ka: Thousand years
Ma: Million years
SNPs: Single nucleotide polymorphisms
UCEs: Ultraconserved Elements
