# Supplementary material for: Range-wide population genomics of common seadragons shows secondary contact over a former barrier and insights on illegal capture
Source: BMC Biol. 2023 May 29;21:129. doi: 10.1186/s12915-023-01628-9 (PMC10228089; doi:10.1186/s12915-023-01628-9)
Supplement: Supplementary file 1 — Additional file 1: Fig. S1. Multi-individual species trees for 268 individuals of leafy, ruby and common seadragons based on 891,858 base pairs including 13,748 SNPs. Fig. S2. Phylogenetic relationships based on mitochondrial genomesof 155 individuals of leafy, ruby and common seadragons. Fig. S3. Cross entropy values for 20 replicates of Admixture analyses, each runs for 1–15 ancestral populations. Fig. S4. Results of individual clustering of 198 individuals with Admixture. Fig. S5. Pairwise FST comparisons between populations. Fig. S6. Estimates of D statistics between pairs of populations. Fig. S7. Overview of the scenarios tested in DIYABC Random Forest. Fig. S8. Comparison of metrics of genetic differentiation and genetic diversity between the present study and the previous study by Klanten et al. 2020. Table S1. Sampling information for the 198 individuals of common seadragon. Table S2. Priors for the DIYABC Random Forest analysis. Table S3. Assignment of two confiscated samples based on population assignment in GenoDive using likelihood ratios. [file 12915_2023_1628_MOESM1_ESM.pdf]

Additional file 1: Supplementary Figures S1-S7 and Tables S1-S3.

Fig. S1: Multi-individual species trees for 268 individuals of leafy, ruby and common seadragons based on 891,858 base pairs including 13,748 SNPs.
Fig. S2: Phylogenetic relationships based on mitochondrial genomes (14,075 base pairs) of 155 individuals of leafy, ruby and common seadragons.
Fig. S3: Cross entropy values for 20 replicates of Admixture analyses, each runs for 1-15 ancestral populations (K).
Fig. S4: Results of individual clustering of 198 individuals with Admixture.
Fig. S5: Pairwise FST comparisons between populations.
Fig. S6: Estimates of D statistics between pairs of populations.
Fig. S7: Overview of the scenarios tested in DIYABC-RF.
Fig. S8: Comparison of metrics of genetic differentiation and genetic diversity between the present study and the previous study by Klanten et al. 2020.
Table S1: Sampling information for the 198 individuals of common seadragon.
Table S2: Priors for the DIYABC-RF analysis.
Table S3: Assignment of two confiscated samples based on population assignment in GenoDive using likelihood ratios.

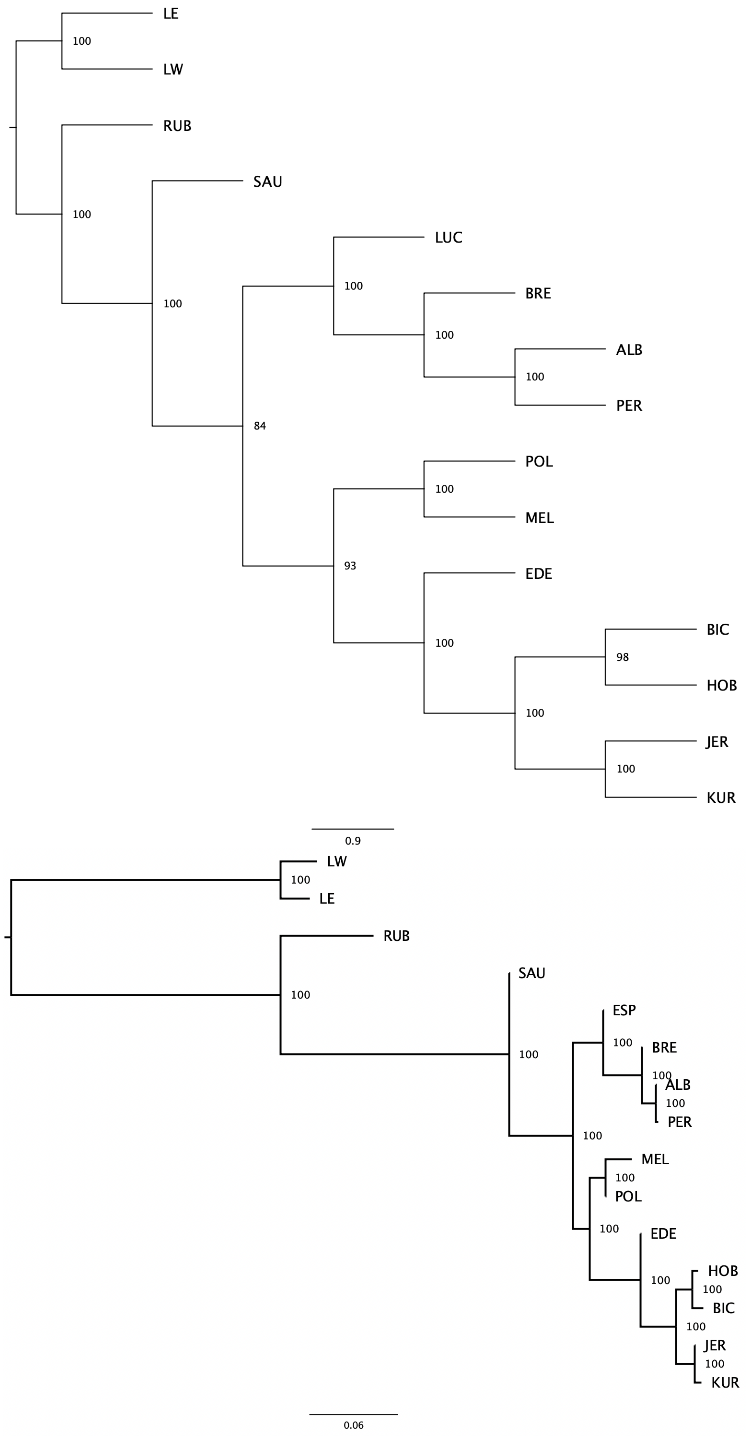

Fig. S1: Multi-individual species trees for 268 individuals of leafy, ruby and common seadragons based on 891,858 base pairs including 13,748 SNPs. *Top*: Tropology from SVDquartets. *Bottom*: Topology from PoMo under the model selected with ModelFinder (TVM+F+P+N9+G4, chosen according to BIC).

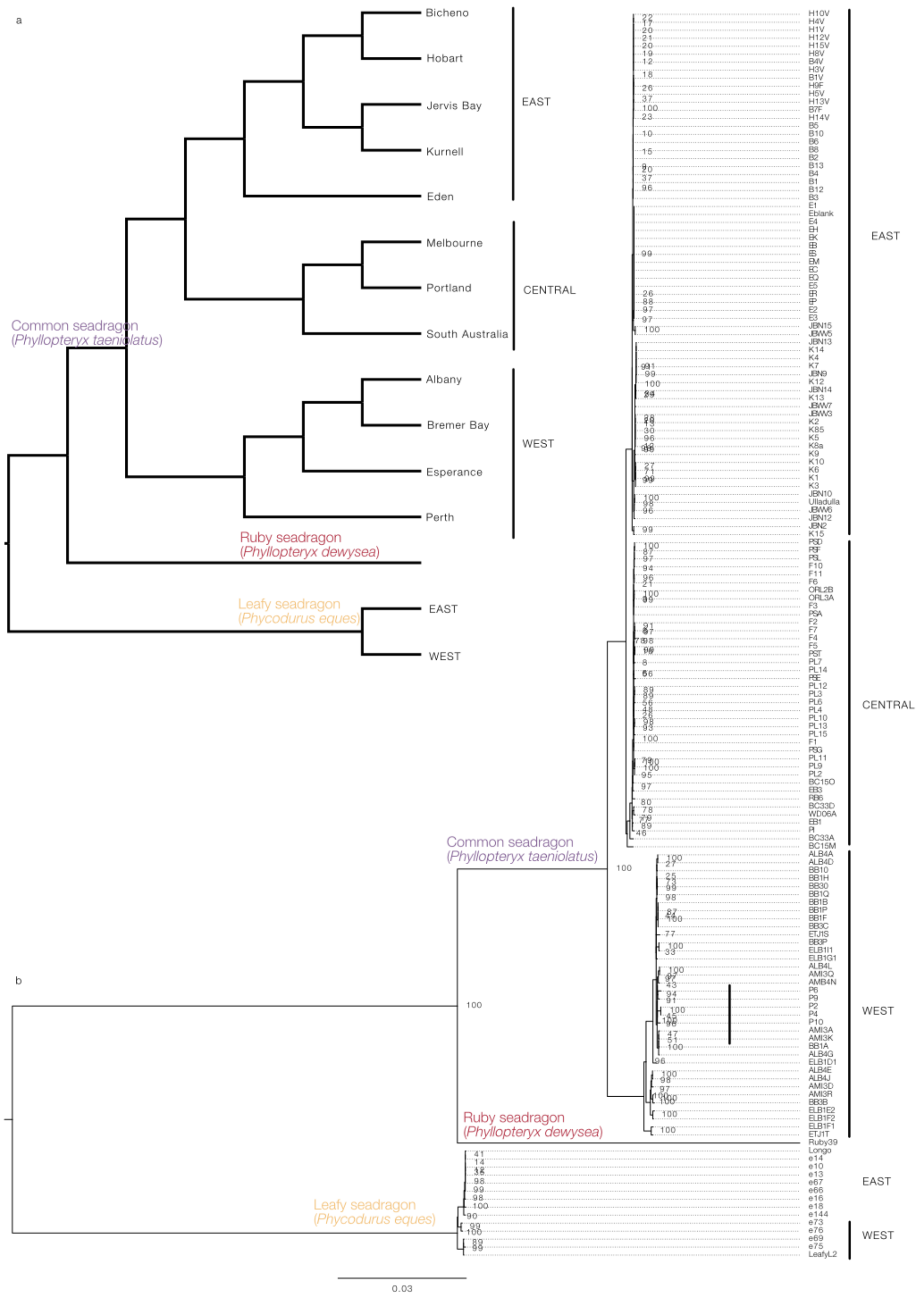

Fig. S2: Phylogenetic relationships based on mitochondrial genomes (14,075 base pairs) of 155 individuals of leafy, ruby and common seadragons. **(a)** Topology from the multi-individual Bayesian species tree analysis STARBEAST2. The tree is the same as in Fig. 2a but is showing the topology to better visualize the branching order in common seadragons. **(b)** Maximum likelihood phylogenetic tree keeping all individuals as separate branches.

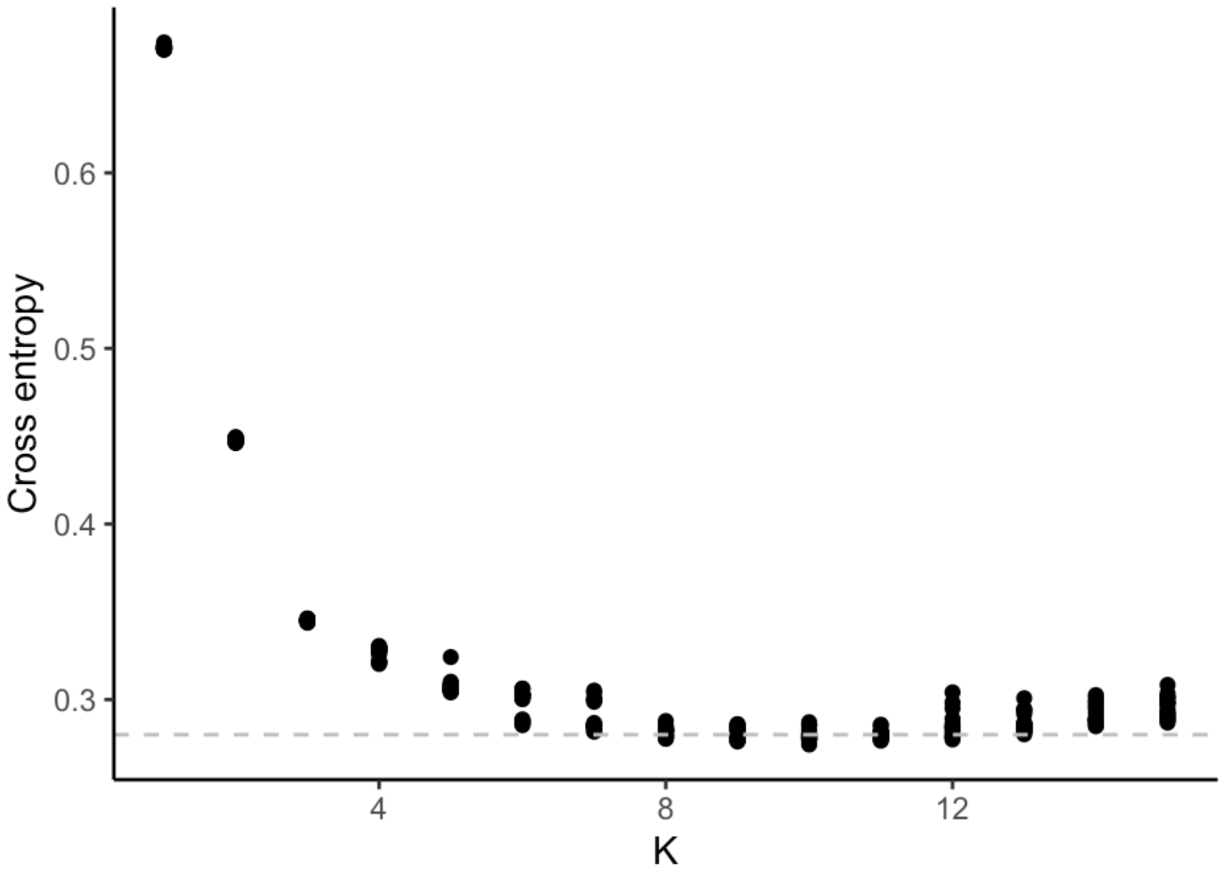

Fig. S3: Cross entropy values for 20 replicates of Admixture analyses, each runs for 1-15 ancestral populations (K). There was no clear lowest value of cross entropy, indicating complex population structure across many values of K. No further improvement of cross entropy past K=8, which was chosen as the highest value of K shown.

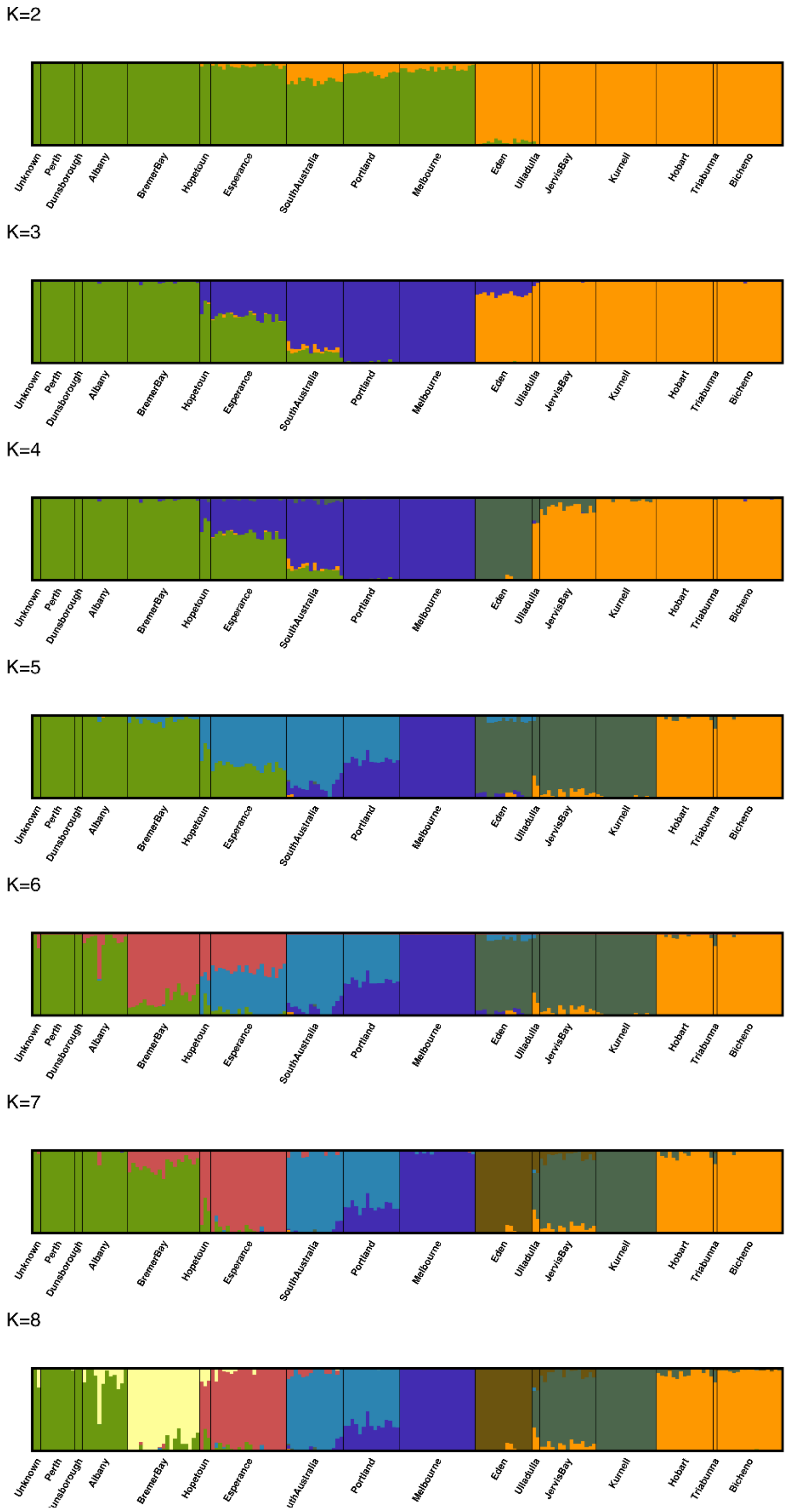
K=2
K=3
K=4
K=5
K=6
K=7
K=8
Unknown
Perth
Dunsborough
Albany
BremerBay
Hopetoun
Esperance
SouthAustralia
Portland
Melbourne
Eden
Ulladulla
JervisBay
Kurnell
Hobart
Triabunna
Bicheno

K=9

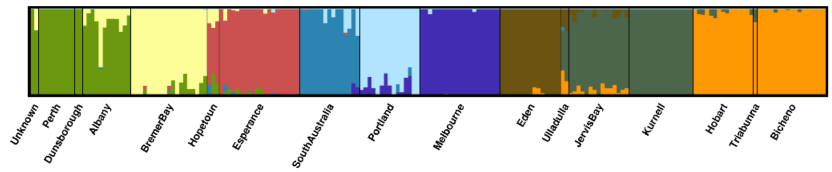

K=10

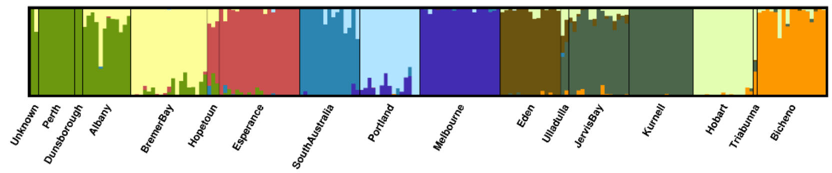

K=11

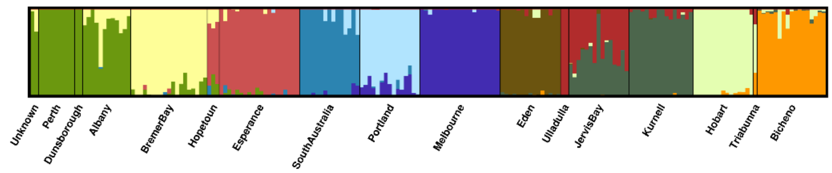

K=12

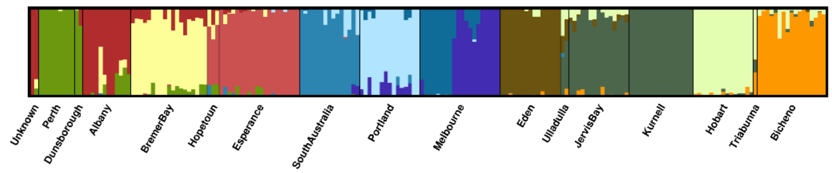

K=13

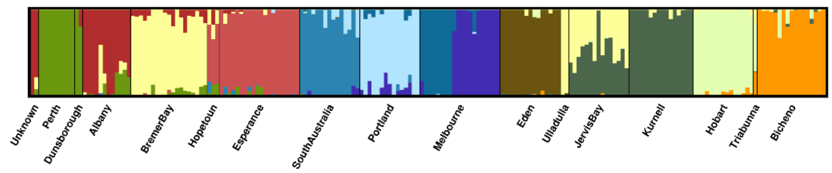

K=14

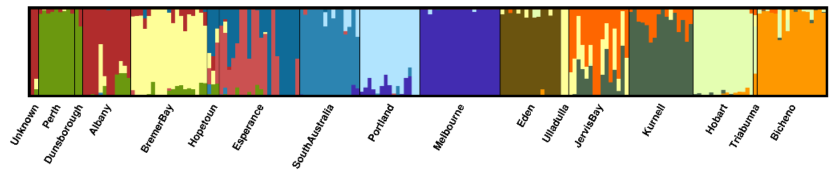

K=15

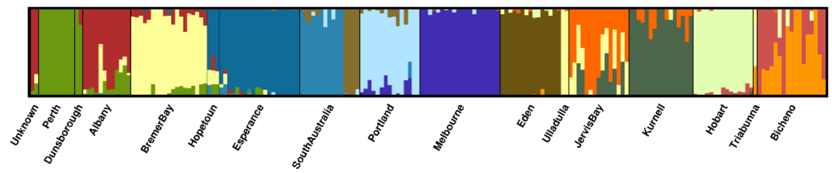

Fig. S4: (Previous two pages) Results of individual clustering of 198 individuals with Admixture. Runs were run for 20 replicates for 1-15 ancestral populations (K) and summarized using Clumpak.

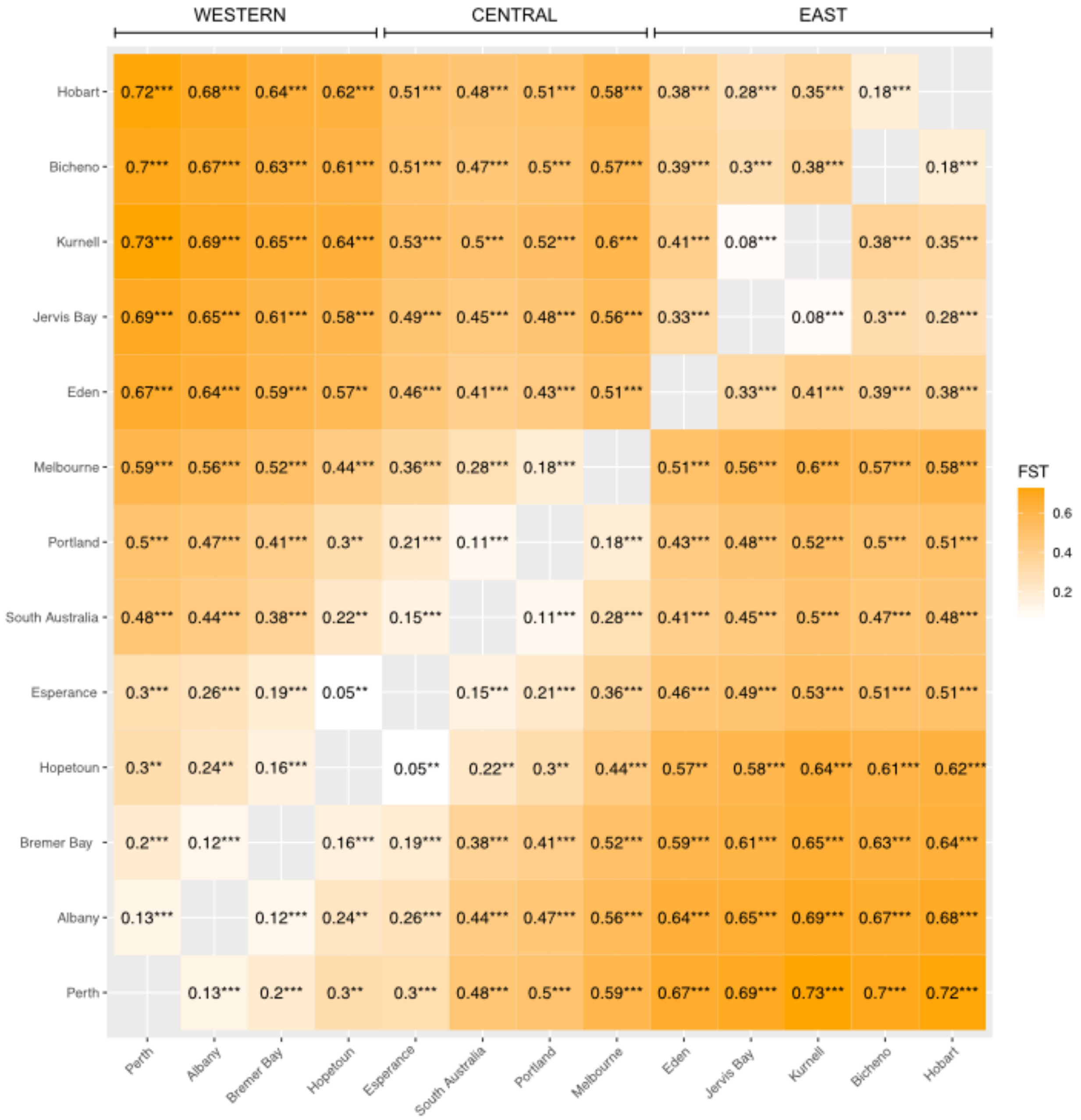

Fig. S5: Pairwise F_{ST} comparisons between populations. Populations are arranged from west to east. Significance was assessed using 1000 permutations. All comparisons were highly statistically significant (*** $p < 0.001$), with the exception of some comparisons with the Hopetoun population, which only had a sample size of N=3 (** $p < 0.01$).

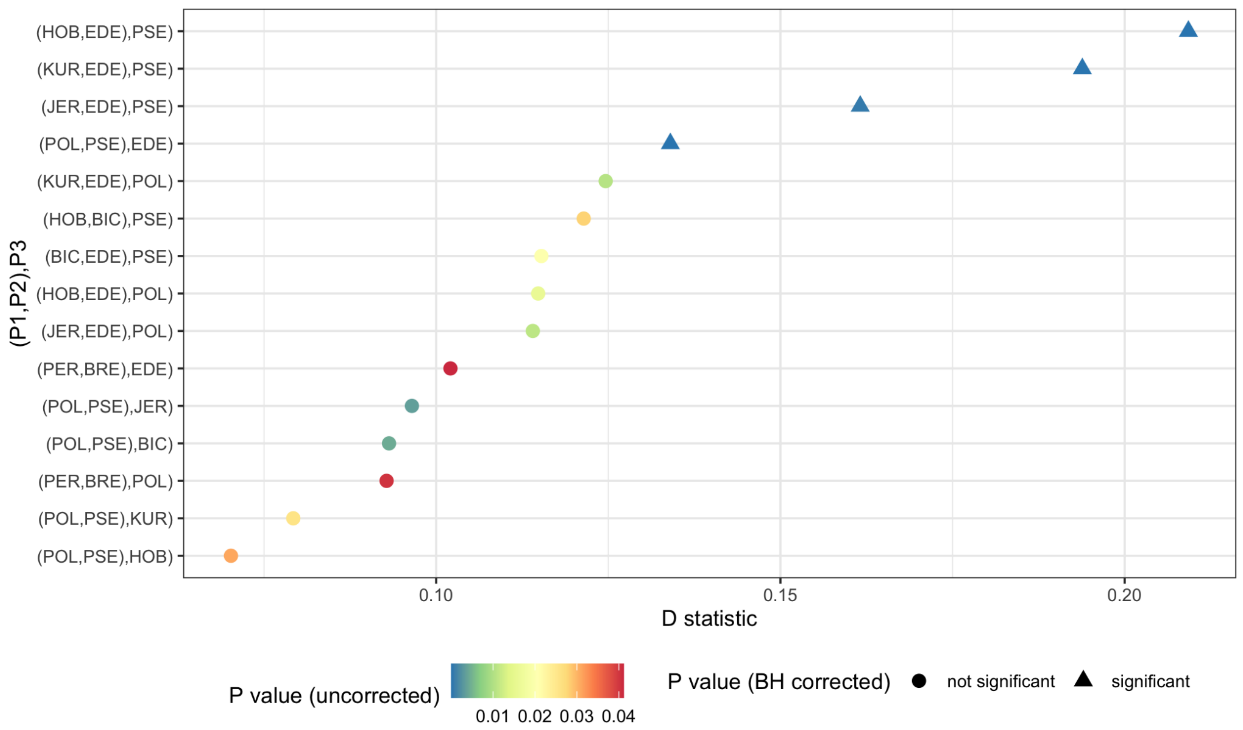

Fig. S6: Estimates of D statistics between pairs of populations. Of all pairwise comparisons between populations, only comparisons that were statistically significant at $p < 0.05$ are shown. All these comparisons involve populations on the east coast and the Victorian sites in the central group. When the value is adjusted for multiple testing (BH method), four comparisons with the highest values for D remain statistically significant (p adjusted < 0.05) as indicated by the triangles (Table 1).

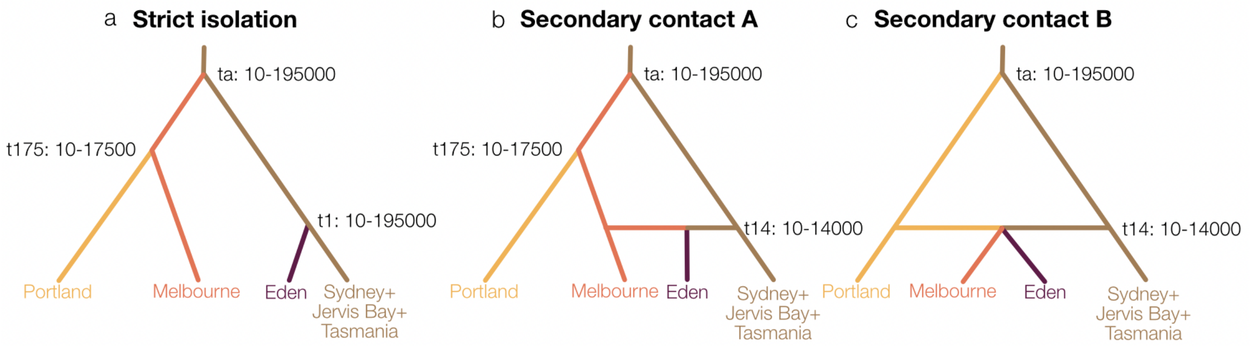

Fig. S7: Overview of the scenarios tested in DIYABC-RF. Values indicate prior distribution for parameters. Scenario b was selected as the best supported scenario.

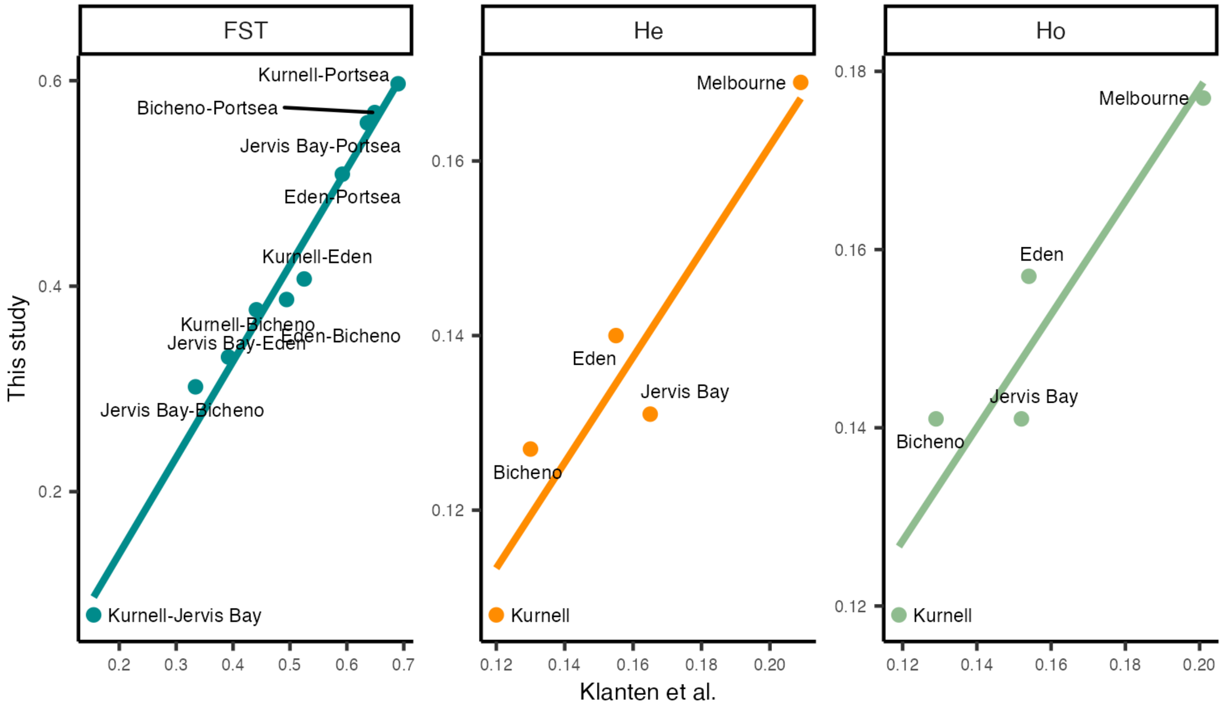

Fig. S8: Comparison of metrics of genetic differentiation and genetic diversity between the present study and the previous study by Klanten et al. 2020. This is limited to the populations sampled by Klanten et al. 2020 (populations of the eastern group and one population of the central group), where we could draw direct comparisons to our sampled populations. Both studies employed different genetic loci (this study: UCEs, Klanten et al.: RADseq) sequenced across different individuals. Yet, the overall patterns are congruent, indicating that the type of genetic loci sequenced does not cause qualitative differences in the estimates of differentiation and genetic diversity.

Table S1: Sampling information for the 198 individuals of common seadragon. N number of samples for each locality.

Locality: Site		N	Collection date	Coordinates	Individual codes	Voucher / tissue number
	Unknown (confiscated, illegally caught from unknown location)	2				
Western Australia						
	Perth	8	13-Mar-2008	31.99 S 115.73 E	P2, P3, P4, P5, P6, P7, P9, P10	-
	Perth: Rottnest Island	1			WAM34845	WAM34845
	Dunsborough: Shelley Cove	2	17-Dec-2005	33.54 S 115.03 E	AB84, Shjuv	ABTC 85684; -
	Albany: Misery Beach	3	15-Dec-2005	35.09 S 117.96 E	AMB4G, AMB4L, AMB4N	-
	Albany: Mistaken Island	5	14-Dec-2005	35.06 S 117.94 E	AMI3A, AMI3D, AMI3K, AMI3Q, AMI3R	-
	Albany: Little Beach	4	15-Dec-2005	34.97 S 118.20 E	ALB4A, ALB4D, ALB4E, ALB4J	-
	Bremer Bay: Back Beach Bommie	10	11 - 13-Dec-2005	34.41 S 119.39 E	BB1A, BB1B, BB1F, BB1H, BB1O, BB1P, BB1Q, BB3B, BB3C, BB3O	-
	Bremer Bay: Little Boat Harbor	6	11-Apr-2016	34.43 S 119.40 E	S24112, S24113, S24114, S24116, S24127, S24129	WAM16-053, WAM16-054, WAM16-055, WAM16-056, WAM16-057, WAM16-059
	Bremer Bay: beach-washed	3	Unknown	Beach-washed; 34.41 S 119.39 E	WAM004, WAM005, WAM007	WAM P.034300-004, P.034300-005, P.034300-007
	Hopetoun: beach-washed	3	14, 19-Feb-2016, 3-Mar-2016	33.94 S 120.11 E	S24108, S24109, S24110	WAM P.34607-001, P.34608-001, P.34609-002
	Esperance: Tanker Jetty	1	10-Dec-2005	33.85 S 121.90 E	ETJ1T	-

	Esperance: Lucky Bay	11	9, 10-Dec-2005	33.99 S 122.22 E	ELB1A1, ELB1C1, ELB1D1, ELB1E2, ELB1F1, ELB1F2, ELB1G1, ELB1I1, ELB1J1, ELB1J2, ELB1S	-
	Esperance: Lucky Bay	8	30-Mar-2016, 2-Apr-2016	33.99 S 122.22 E	S24071, S24072, S24074, S24076, S24081, S24085, S24086, S24087	WAM16-009, WAM16-010, WAM16-012, WAM16-014, WAM16-020, WAM16-023, WAM16-024, WAM16-025
South Australia						
	Pearson Island	1	24-May-2006	33.96 S 134.26 E	PI	-
	Spencer Gulf: Station BC15	2	17-Feb-2007	34.10 S, 137.02 E	BC15M, BC15Q	SAMA F 14316, F 14320
	Spencer Gulf: Station BC33	3	16-Feb-2007	34.60 S 137.38 E	BC33A, BC33B, BC33D	SAMA F 14324, F 14325, F 14327
	Spencer Gulf: Station WD06	4	16-Feb-2007	34.39 S 137.37 E	WD06A - D	SAMA F 14330, F 14331, F 14332, F 14333
	Spencer Gulf: Station Z3/10	1	19-Feb-2007	34.01 S 136.88 E	Z310A	SAMA F 4302
	Fleurieu Peninsula: Rapid Bay	1	28-May-2006	35.52 S 138.19 E	RB6	-
	Fleurieu Peninsula: Encounter Bay	2	8-Apr-2006	35.58 S 138.62 E	EB1, EB3	-
	Fleurieu Peninsula: Goolwa beach-washed	1	1-Aug-2005	Beach-washed; 35.52 S 138.74 E	84486	-
Victoria						
	Portland	15	25, 26-Mar-2007	38.34 S 141.61 E	PL1, PL2, PL3, PL4, PL5, PL6, PL7, PL8, PL9, PL10, PL11, PL12, PL13, PL14, PL15	-
	Melbourne: Portsea	8	28-Mar-2007	38.32 S 144.71 E	PSA, PSD, PSE, PSF, PSG, PSJ, PSL, PST	-

	Melbourne: Flinders	10	22-Aug-2006	38.48 S 145.03 E	F1, F2, F3, F4, F5, F6, F7, F8, F10, F11	-
	Melbourne: Flinders (Captive)	2	Unknown		ORL2A, ORL3B	-
Tasmania						
	Bicheno	17	16 - 18-Mar-2007	41.87 S 148.30 E	B1V, B4V, B5V, B7F, B8V, H10V, H11V, H12V, H13V, H14V, H15V, H1V, H3V, H4V, H5V, H8V, H9F	-
	Triabunna: Okehampton Beach	1	29-Aug-2009	42.51 S 147.97 E	T8296	CSIRO H 7758-01, GT 8296
	Hobart: Frederick Henry Bay, Cremorne Beach	1	10-Nov-2005	42.97 S 147.53 E	T122	CSIRO H 6291-01, GT 122
	Hobart: Blackmans Bay	1	14-Mar-2007	42.98 S 147.32 E	H10-Fpot	-
	Hobart: Blackmans Bay	7	15, 18 - 19-Dec-2012	42.98 S 147.32 E	B1, B2, B8, B9, B10, B12, B13	-
	Hobart: Mirramar Park	4	16-Dec-2012, 18-Dec-2012	42.99 S 147.33 E	B3, B4, B5, B11	-
	Hobart: Kingston Beach	2	17-Dec-2012	43.00 S 147.33 E	B6, B7	-
New South Wales						
	Eden	15	3, 4-Apr-2007	37.08 S 149.91 E	E1, E2, E3, E4, E5, EB, EC, EH, EK, EM, EP, EQ, ER, ES, Eblank	-
	Bawley Point/Ulladulla: Bawley Point	1	Unknown	35.52 S 150.40 E	t17	ABTC 85017
	Bawley Point/Ulladulla: Ulladulla	1	Unknown	35.37 S 150.49 E	Longo et al. (2017)	AMS I.45022-030
	Jervis Bay: Nursery	10	5-Apr-2007	35.03 S 150.69 E	JBN1, JBN2, JBN8, JBN9, JBN10, JBN11, JBN12, JBN13, JBN14, JBN15	-
	Jervis Bay: Weedy Valley	5	5-Apr-2007	35.03 S 150.69 E	JBWV3, JBWV4, JBWV5, JBWV6, JBWV7	-

	Kurnell: Site 1	13	9-Apr-2007	34.01 S<br>151.21 E	K1, K2, K3, K4, K5, K6, K7, K85, K9, K10, K11, K14, K15	-
	Kurnell: Site 2	3	9-Apr-2007	34.01 S<br>151.21 E	K12, K13, K8a	.

Abbreviations: ABTC Australian Biological Tissue Collection, South Australian Museum, Adelaide; AMS Australian Museum, Sydney; CSIRO Australian National Fish Collection, Hobart, CSIRO National Facilities and Collections; SAMA F South Australian Museum, Fish Collection, Adelaide; SARDI South Australian Research and Development Institute Aquatic Sciences; WAM Western Australian Museum, Perth

Table S2: Priors for the DIYABC-RF analysis. The right side of the table gives the posterior estimates for the parameters of the best scenario (scenario 2). Parameter t1 was not used in this scenario and therefore has no posterior estimates.

		Prior parameters		Posterior estimates		
Parameter	Parameter description	Minimum	Maximum	Median	Quantile 5%	Quantile 95%
JKT	Population size of Jervis Bay, Kurnell, Hobart and Bicheno	1000	1000000	79616	37793	232206
EDE	Population size of Eden	1000	1000000	13974	1854	748915
MEL	Population size of Melbourne	1000	1000000	12307	3885	35885
POL	Population size of Portland	1000	1000000	614920	286916	963599
t_{14}	Fusion time of Melbourne and Eden	10	14000	7376	1731	13015
t_{175}	Divergence time of Portland and Melbourne	10	17500	10284	3321	16884
t_1	Divergence time of Eden and remaining east coast populations	10	195000	NA	NA	NA
t_a	Divergence time of the central and eastern group	10	195000	145918	71941	190782
hy	Admixture rate	0.01	0.99	0.83	0.52	0.94
Temporal constraints: $t_a>t_{175}>t_{14}>t_1$						

Table S3: Assignment of two confiscated samples based on population assignment in GenoDive using likelihood ratios. Assignments for Hopetoun could not be calculated because too few reference individuals were available (N=3). The locality with the highest likelihood is highlighted in bold.

Population	Individual 1	Individual 2
Perth	-630.747	-515.646
**Albany**	**-353.461**	**-313.398**
Bremer Bay	-500.557	-399.230
Hopetoun	N/A	N/A
Esperance	-876.967	-683.607
South Australia	-1543.846	-1189.057
Portland	-1840.450	-1404.065
Melbourne	-2129.779	-1617.322
Eden	-2670.724	-2080.929
Jervis	-2734.781	-2088.299
Kurnell	-2964.725	-2289.722
Bicheno	-2847.156	-2237.744
Hobart	-2895.284	-2315.154

Additional file 2: Sequencing statistics, mapping and deduplication statistics and SRA accessions for each sequencing run (sheet 1), each sample (sheet 2). Genbank accessions for the mitochondrial genomes (sheet 3). (XLSX format)
